# Mortality Trends in Patients Hospitalized with the Initial Acute Myocardial Infarction in a Middle Eastern Country over 20 Years

**DOI:** 10.1155/2014/464323

**Published:** 2014-04-28

**Authors:** Emad Ahmed, Jassim Al Suwaidi, Ayman El-Menyar, Hajar A. H. AlBinali, Rajvir Singh, A. A. Gehani

**Affiliations:** ^1^Department of Adult Cardiology and Cardiovascular Surgery, Heart Hospital, P.O. Box 3050, Doha, Qatar; ^2^Department of Cardiology, National Heart Institute, Cairo, Egypt; ^3^Department of Clinical Medicine, Weill Cornell Medical School, P.O. Box 24144, Doha, Qatar; ^4^Clinical Research, Trauma Section, Hamad Medical Corporation (HMC), P.O. Box 3050, Doha, Qatar; ^5^Internal Medicine, Cardiology Section, Ahmed Maher Teaching Hospital, Egypt

## Abstract

We aimed to define the temporal trend in the initial Acute Myocardial Infarction (AMI) management and outcome during the last two decades in a Middle Eastern country. A total of 10,915 patients were admitted with initial AMI with mean age of 53 ± 11.8 years. Comparing the two decades (1991–2000) to (2001–2010), the use of antiplatelet drugs increased from 84% to 95%, **β**-blockers increased from 38% to 56%, and angiotensin converting enzyme inhibitors (ACEI) increased from 12% to 36% (*P* < 0.001 for all). The rates of PCI increased from 2.5% to 14.6% and thrombolytic therapy decreased from 71% to 65% (*P* < 0.001 for all). While the rate of hospitalization with Initial MI increased from 34% to 66%, and the average length of hospital stay decreased from 6.4 ± 3 to 4.6 ± 3, all hospital outcomes parameters improved significantly including a 39% reduction in in-hospital Mortality. Multivariate logistic regression analysis showed that higher utilization of antiplatelet drugs, **β**-blockers, and ACEI were the main contributors to better hospital outcomes. Over the study period, there was a significant increase in the hospitalization rate in patients presenting with initial AMI. Evidence-based medical therapies appear to be associated with a substantial improvement in outcome and in-hospital mortality.

## 1. Introduction

Cardiovascular (CV) disease is a leading cause of morbidity and mortality worldwide. The epidemiology of coronary artery disease (CAD) is known to undergo changes over time. Some reflect changes in risk factors or management, while others are difficult to explain. What is certain is that changes in the rate and mortality from CAD have a major impact on the overall health care system [1].

Guidelines from major cardiac societies [2–4] strongly support the use of *β*-blockers (BB) and antiplatelet agents in patients who survive AMI. Several recent clinical trials [5] have also demonstrated the beneficial effects associated with the use of angiotensin converting enzyme inhibitors (ACEI) in patients with acute coronary syndrome (ACS), irrespective of the extent of left ventricular dysfunction.

The management of AMI has undergone impressive changes during the last 2 decades [2] and CAD mortality has also declined in many countries [6]. This decline in CAD mortality is partially related to a decrease in both the rate and the case fatality of this disease [7–9]. While reduced CAD M fatality is primarily related to the use of effective treatments [10–12], the decreased incident of CAD is largely explained by CV risk factors modification [13, 14].

In the present study, we analyzed the temporal trends in hospitalization rate, in-hospital mortality, and changes in the utilization of evidence-based therapies early in the course of initial AMI (both ST-elevation and non-ST-elevation). The association between such changes and the hospital outcomes were studied across two different time periods (1991–2000 versus 2001–2010).

## 2. Methods

### 2.1. Study Setting

We conducted this cohort study in Qatar, which is a country in the Middle East. The population was around 600,000 (2001 Census) and 1.6 million (2010 Census). This study was based at the Cardiology service at Hamad Medical Corporation, Doha, Qatar, which provides the main inpatient and outpatient tertiary care for the whole country and for the residents in Qatar (nationals and expatriates). More than 95% of cardiac patients are treated in this hospital making it an ideal center for population-based studies. The Cardiology Database is maintained electronically from January 1991, and data up to December 2010 was used for present study. The data forms were filled by the Cardiologists at the time of patient discharge from the hospital according to predefined criteria for each data point. These records are coded, registered, and entered into a computer by a data entry operator and are randomly checked by the cardiology department [15]. The 20-years-period was divided into 2 inclusive 10-year periods, (1991–2000 and 2001–2010). Information on management variables including cardiac medications, coronary reperfusion therapy [thrombolytic therapy, percutaneous coronary intervention (PCI), or coronary artery bypass grafting (CABG)], and all in-hospital clinical complications and mortality was collected. Patients with incomplete data were excluded. The primary outcome of this analysis was all-cause in-hospital mortality while secondary outcomes included rates of in-hospital complications, such as resuscitated cardiac arrest, congestive heart failure (CHF), cardiogenic shock, and cerebrovascular accident (CVA). Ethical approval was obtained from MRC Research Committee, for the analysis and publication of the study.

## 3. Definitions

Initial MI was defined as AMI in a patient who denied pervious history of MI and the medical record did not show an evidence of previous MI.

### 3.1. Traditional Risk Factors

Diabetes mellitus (DM), hypertension, and dyslipidemia were identified when patients were known to have the given risk factor(s) prior to the index admission and/or were already on treatment for that condition. The presence of DM was determined by the documentation or diagnosis of DM that had been treated with medications or insulin. The presence of hypertension was determined by any documentation in the medical record of hypertension and/or if the patient was already on treatment by a physician. The presence of dyslipidemia was determined by the demonstration of a fasting cholesterol > 5.2 mmol/L in the patient's medical record and/or history of treatment of dyslipidemia.

### 3.2. Smoking History

Patients were defined as smoking any form of tobacco and divided into nonsmokers, current cigarette smokers, or past smokers (defined as more than 6-month abstinence). Chronic renal impairment was defined as serum creatinine that is >1.5 times the upper normal range. Congestive heart failure (CHF) was defined using the Framingham criteria. Minor criteria were acceptable only if they could not be attributed to any other medical condition (such as chronic lung disease, cirrhosis, ascites, or the nephrotic syndrome) [16]. Family history of premature CAD was defined as any direct blood relatives (parents, siblings, and children) who have had any of the following at 55 years or younger: angina, MI, or sudden cardiac death without obvious cause.

### 3.3. Statistical Analysis

Data were presented in the form of frequency and percentages for categorical variables and mean ± standard deviation (SD) for interval variables. Baseline demographic characteristics, past medical history, clinical presentation, medical therapy, cardiac procedures, and in-hospital clinical outcomes of initial acute myocardial infarction (AMI) were analyzed across two inclusive ten-year periods from 1991–2000 to 2001–2010. Statistical analyses were performed using student *t*-tests or Wilcoxon Rank sum tests for interval variables between the two groups wherever applicable. Pearson chi-square (*χ* ^2^) tests were applied for categorical variables. Trends in cardiac medications with *β*-blockers, antiplatelets, and ACE inhibitors within 24 hours after presentation and at discharge were assessed. Also, trends in in-hospital mortality according to gender, age group, and type of the initial AMI were presented for every five-year period. Variables influencing in-hospital mortality (age, sex, risk factors, admission medications, and procedures) were assessed with multiple logistic regression analysis (enter method). Adjusted odds ratios (OR), 95% CI, and *P* values were reported for significant predictors for all and each group separately. All *P* values were the results of 2-tailed tests and values <0.05 were considered statistically significant. Statistical Package for Social Sciences (SPSS) version 19.0 has been used for the analysis.

## 4. Results

### 4.1. Study Population

Between 1991 and the end of 2010, a total of 41,438 patients were hospitalized with acute cardiac diseases; of these, 10,915 (26.3%) consecutive patients fitted the definition of initial AMI. The 20-year study period was divided into 2 inclusive 10-year periods. The characteristics of AMI patients across the study periods are shown in Table 1.

#### 4.1.1. Trends in Patient Characteristics

Over the time periods studied, theoverall number of initial AMI hospitalization from total hospitalization increased from 3,740 (34%) in 1991–2000 to 7,175 (66%) in 2001–2010.

*(i) Trends in Age, Gender, Ethnicity, and Initial AMI. *Overall, the mean age of the study populations increased from 51 ± 12 to 54 ± 12 years (*P* for trend <0.001). There was a significant decline in the rate of admission for younger patient age group (≤50 years) from 53% to 43% but the proportion of elderly patients (>70 years) increased from 6.0% to 9.0% (all *P* for trend <0.001). The proportion of South Asian (SA) to other ethnicities and the ratio of men to women ratio did not change significantly. Nearly 50% of the study population was from SA and men accounted for the majority (88%).

*(ii) Trends of the Cardiovascular Risk Factors*. With respect to cardiovascular risk factors, the rates of obesity, current smoking, hypertension (HTN), diabetes mellitus (DM), and chronic renal impairment increased significantly (all *P* for trend <0.001). Also, the body mass index (BMI) increased from 25 ± 4 to 27 ± 6 (mean ± SD) (all *P* for trend <0.001). However, family history of CAD did not change (*P* < 0.34), while dyslipidemia decreased over time from 26% to 18% (*P* for trend <0.001). Although the total serum cholesterol and triglyceride (TG) levels decreased from 5.4 ± 12 to 5.0 ± 1.3 and from 2.04 ± 1.2 to 1.9 ± 1.16, respectively, (*P* for trend <0.001) the mean HDL-C level did not change over the study period (1.03 ± 0.23 to 1.02 ± 0.31, *P* for trend <0.73).

#### 4.1.2. Trends in Management

**Figure 1:**
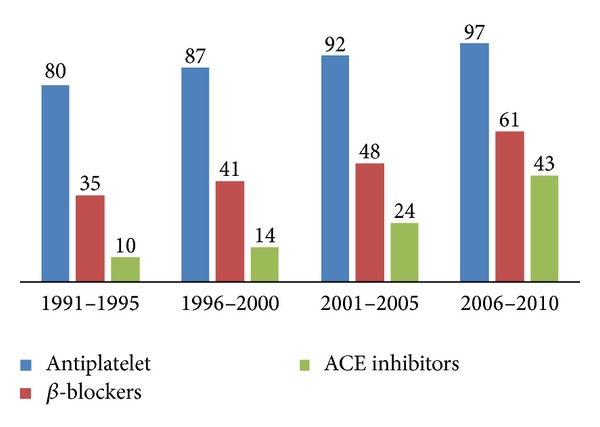
Trends in hospital medications (%) over 2 decades.

*Trends in the Use of Medical Therapies, Coronary Reperfusion, and Revascularization Procedures.* In the entire study population, a significant increase in the use of certain CV medications during hospitalization was observed over the span of 20 years including marked increases in the use of ACE inhibitors (from 12% to 36%) and *β*-blockers (from 38% to 56%). Less but significant increase was also noted in the use of antiplatelet drugs (from 84% to 95%) and in the use of heparins (unfractionated and LMWH) (from 56% to 59%) (all *P* for trend <0.001) (Table 2) (Figure 1).

**Figure 2:**
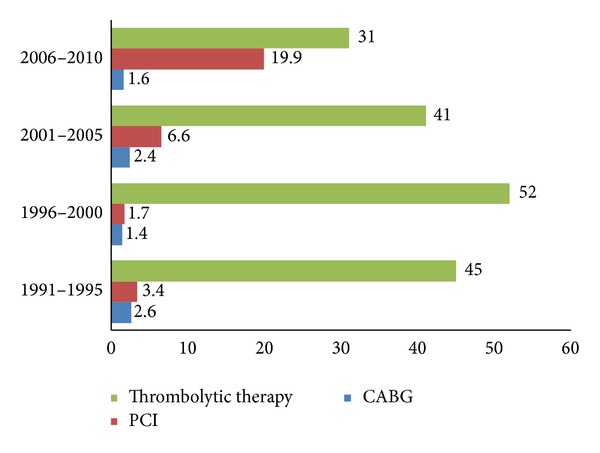
Trends in coronary reperfusion and revascularization procedures (%) over 2 decades.

During the last two decades, thrombolytic therapy was used as a primary reperfusion therapy; however there was a substantial change in the proportion of patients who were treated with PCI. The rate of thrombolytic therapy administration declined from 71% to 65% while the rate of PCI increased from 2.5% to 14.6% (*P* < 0.001). Finally, no significant trends were found forthe rate ofCABG (*P* for trend <0.99) (Figure 2). 

*(i) Medication at Discharge.* At discharge, there was a significant increase in the use of evidence based CV medications including ACE inhibitors (from 12% to 36%), *β*-blockers (from 38% to 56%), and antiplatelet drugs (from 89% to 97%), *P* for trend <0.001 for all. 

*Trends in Hospital Length of Stay and Outcomes (Table 2).*  Although there was no significant change between the 2 decades regarding the length of stay in the coronary care unit (3 ± 1.7 versus 3 ± 1.6 days (*P* for trend <0.40), the total hospital stay was reduced by 28%, from 6.4 ± 3 days to 4.6 ± 3 days (*P* < 0.001).

During the study period, the overall in-hospital mortality had substantially decreased from 8.8% (in 1991–2000) to 5.4% (in 2001–2010), representing a 38.6% relative reduction. This was observed in all age groups and in both genders. However, female gender and the elderly (>70 years) were associated with higher mortality than their counterparts, males and younger patients (<70 years), respectively. Also, the overall rates of in-hospital complications declined significantly, including cardiac arrest, CHF, cardiogenic shock, and CVA (all *P* for trend <0.001).

### 4.2. Multivariate Logistic Regression Analysis (Table 3)

We used logistic regression analysis to assess the mortality among patients hospitalized for initial AMI with adjustment for baseline variables.

The main predictors of in-hospital mortality included age (>70 years) (adjusted OR: 3.60, 95% CI: 2.74–4.63, *P* < 0.001), DM (adjusted OR: 1.8, 95% CI: 1.81–2.15, *P* < 0.001), and female gender (adjusted OR: 1.70, 95% CI: 1.39–2.13, *P* < 0.001). Moreover, hospital therapies were associated with lower in-hospital mortality: antiplatelet drugs (adjusted OR: 0.20; 95% CI: 0.16–0.26); *β*-blockers (adjusted OR: 0.28; 95% CI: 0.23–0.34); ACE inhibitors (adjusted OR: 0.37; 95% CI: 0.30–0.48); and reperfusion therapy (adjusted OR: 0.66; 95% CI: 0.47–0.98). The use of antiplatelet drugs, *β*-blockers, ACEI, and PCI was associated with a reduction in the in-hospital mortality rate by 80%, 72%, 63%, and 34%, respectively (Table 3).

The age-adjusted in-hospital mortality rate was higher in females compared with their counterpart males in all age groups. The difference in mortality between genders after initial AMI decreased with age (relative risk ranging from 4.0 (95% CI: 2.56–6.25) at ≤50 years to 2.27 (95% CI: 1.79–2.94) at 51–70 years and 1.46 (95% CI: 1.04–2.06) at >70 years).

## 5. Discussion

Our results provide several important insights into the population trends associated with initial AMI. (1) CAD risk factors control is still insufficient, which could largely account for the high burden and rate of AMI admissions. (2) Treatment with evidence based therapies increased over time and probably played a major role in shorter hospital stay and significant improvement in hospital outcomes of both mortality and morbidity. (3) Despite these encouraging trends, there is an opportunity for better control of risk factors and more optimal use of evidence based therapies.

### 5.1. Baseline Characteristics

From decade to decade, we noticed increased mean age among patients hospitalized with initial AMI which may suggest improvement in primary prevention of CAD throughout the last decade. The overall age in the entire study period was 52 years, which is significantly younger by almost 10 years when compared with reports from the developed countries [17, 18]. This finding may be contributed to South Asians by a higher proportion,, a population known to be prone to develop CAD at a younger age and increased CV risk factor burden [19–22]. The latter may have also contributed to increased rate of initial AMI [23]. An increased proportion of CV risk factors in patients with AMI suggests further opportunities for aggressive screening and risk factors modification and reinforces the importance of preventive measures by lifestyle advice and drugs.

Previous studies have suggested that the incidence of CAD and associated mortality has declined in many countries [22, 24–27]. Data from MIYAGI-AMI registry study demonstrated that in the last two decades there was a steady trend of increasing incidence of AMI [23].

In the present study, we report an increased rate of AMI hospitalization from 34% to 66% in the last two decades. Explaining the increased rate in initial AMI hospitalization in our study is complex. Part of this increase might be due to change in criteria defining AMI [28]. The increased penetration and utilization of troponin as biomarker in Qatar from 1999 onwards would be expected to lead to an increase in AMI hospitalizations. Our data are also in agreement with prior studies in which the rate of AMI was predominantly of the male gender [29, 30].

We observed a significant improvement in in-hospital mortality and morbidity in all age groups and in both genders. The current study reported 6.5% reduction in overall in-hospital mortality rate which is comparable with other European registries (7% in the Euro heart survey and GRACE) [31, 32].

In an attempt to understand the reasons behind this improvement in hospital outcomes, we carried out further analysis taking into account the different therapies that were provided. From 2001 onward, we demonstrated a significant increase in the utilization of ACE inhibitors and *β*-blockers. Smaller increases were noted in the use of antiplatelet and subtle increase in the use of heparins during the initial 24 hours after admission. Multivariate adjustment suggests that most of the decrease in mortality is probably related to improved quality of treatment according to the guidelines and the revascularization interventions [11, 33].

Previous trials [34–40] have suggested a significant role of some drugs in lowering early CV mortality when used in the acute phase of AMI. In particular, GISSI-3 and ISIS-4 trials demonstrated a marginal but statistically significant mortality reduction when ACE inhibitors were started early and persisted up to one year follow up. The ACC/AHA guidelines recommend the administration of ACE inhibitors within the initial 24 hours of STEMI in the absence of hypotension or known contraindications (class I, level of evidence A) [3]. Several large trials including ISIS-1 MIAMI, TIMI-IIb, and GUSTO-I recommended *β*-blockers early in the setting of AMI. Our findings support the recommendation that both BBs and ACE inhibitors were major contributors to the decreased in-hospital mortality, which is consistent with the published trials [37–42].

Despite this improvement in practice, the overall rate of utilization of some of these drugs is still below the current standards when compared to other larger multinational registries [4], and further efforts are therefore needed to optimize their use. Moreover, the hospital stay is expected to decrease further with a full implementation of the recent primary PCI program in Qatar [43].

Another finding which is consistent with AMIS plus registry [44] was the increase in the proportion of acute MI patients treated by PCI and a reduction in the use of thrombolytic therapy, in our study. However, the increase in PCI procedures observed was considerably low which is in agreement with a recent study in 6 Middle-Eastern countries [45]. Such Improvements in management including increased use of evidence-based therapies may have contributed to the decline in hospital stay shown in this study. Throughout the study period, the rate of CABG remained stable at around 2%, a finding consistent with a recent report in the other countries like USA [46].

### 5.2. Study Strengths and Limitations

This study of trends over 20-year period for the initial AMI hospitalizations must be viewed in the context of a number of changes in the clinical practice in the last 2 decades. The inclusion of all patients hospitalized with initial AMI provides a unique opportunity to closely examine the trends in the therapeutic management of an unselected population of all ages. Each data collection period occurred over 10 years and this approach ensures a large enough sample size and represents strength of this study. To the best of our knowledge this is the first study from the Middle East to address the changes in the management and mortality in patients hospitalized with initial acute MI. It is conducted in a large population and over a long time period. Our study is constrained by limitations which are inherent in all studies of historical, observational design. Inaccuracies in the diagnosis and coding of AMI in routine data are well recognized. This could lead to missing of some data or measurement errors. However, the same methodology was used throughout the study period. Our study focused on in-hospital outcomes, but long-term outcomes were not available.

Finally, in spite of these limitations, the findings of this study are interesting as it compares a cohort of unselected patients hospitalized with initial AMI representing the changes in hospital course, treatment strategies, and patient outcome over 2 decades.

## 6. Conclusion

Over the last two decades, there is a significant improvement in the in-hospital outcomes in patients hospitalized with initial AMI. This parallels a significant increase in the use of evidence based therapies. However, there is a growing burden of risk factors. Efforts are therefore needed to further optimize the management and reduce the burden of risk factors in the Middle East.

## Figures and Tables

**Table 1:** Patients characteristics according to study period (1991–2010).

Variables (%)	1991–2000	2001–2010	*P*-value
Number (%)	3740 (34)	7175 (66)	0.001
Age in year (mean ± SD)	51 ± 12	54 ± 12	0.001
Age groups, years			
≤50	1998 (53)	3074 (43)	0.001 For all
51–70	1510 (40)	3429 (48)	
>70	232 (6)	668 (9)	
Male gender	3316 (89)	6294 (88)	0.21
Ethnicity			
South Asians	1828 (49)	3474 (48)	
Middle East Arabs	1497 (40)	2642 (37)	
Others	415 (11.1)	1059 (14.8)	0.001
Cardiovascular risk factors (%)			
Hypertension	891 (23.8)	2834 (39.5)	0.001
Diabetes mellitus	1169 (31.3)	2967 (41.4)	0.001
Current Smoking	1086 (29)	2999 (42)	0.001
Dyslipidemia	959 (26)	1276 (18)	0.001
Family history of CAD	75 (2)	164 (2.3)	0.34
Body mass index (Kg/m^2^) (mean ± SD)	25 ± 4	27 ± 6	0.001
Obesity	61 (1.6)	288 (4)	0.001
Chronic renal impairment	42 (1.1)	241 (3.4)	0.001
Total cholesterol (mmol/L) (mean ± SD)	5.4 ± 1.2	5 ± 1.3	0.001
High-density lipoprotein cholesterol (mmol/L) (mean ± SD)	1.03 ± 0.23	1.02 ± 0.31	0.73
Serum triglyceride (mmol/L) (mean ± SD)	2.04 ± 1.2	1.9 ± 1.16	0.001
CK-MB (u/L) (mean ± SD)	380 ± 1091	190 ± 570	0.001

CAD: coronary artery disease.

**Table 2:** Management and in-hospital outcomes according to study period (1991–2010).

Variables (%)	1991–2000	2001–2010	*P*-value for trend
Medication during 1st 24 h of admission			
Thrombolytic	1820 (71)	2516 (65)	0.001
Antiplatelet drugs	3122 (84)	6822 (95)	0.001
Heparin	2105 (56)	4249 (59)	0.003
*β*-blockers	1415 (38)	4005 (56)	0.001
ACE inhibitors/ARB	452 (12)	2579 (36)	0.001
Coronary angiography	1161 (31)	1309 (18)	0.001
PCI	95 (2.5)	1051 (14.6)	0.001
CABG	73 (2)	140 (2)	0.99
In-hospital outcomes			
Death	329 (8.8)	385 (5.4)	0.001
Cerebrovascular accident	39 (1)	11 (0.2)	0.001
Cardiogenic shock	138 (3.7)	207 (2.9)	0.02
Cardiac arrest	266 (7.1)	363 (5.1)	0.001
Congestive heart failure	210 (5.6)	252 (3.5)	0.001
Medication at discharge			
Antiplatelet agent	3318 (88.7)	6931 (96.6)	0.001
*β*-blockers	1415 (37.8)	4005 (55.8)	0.001
ACE inhibitors/ARB	452 (12.1)	2579 (35.9)	0.001
Final discharge diagnosis			
ST-elevation MI	2547 (68)	3859 (54)	
Non-ST-elevation MI	1193 (32)	3316 (46)	0.001
Hospital days (mean ± SD)			
Coronary care units stay	3 ± 1.7	3 ± 1.6	0.40
Total hospital stay	6.4 ± 3	4.6 ± 3	0.001

ACE inhibitors/ARB: angiotensin converting enzyme inhibitors/angiotensin receptor blocker; CABG: coronary artery bypass grafting; PCI: percutaneous coronary intervention; MI: myocardial infarction.

**Table 3:** Multivariate regression analysis for predictors of in-hospital mortality.

Variables	Adjusted OR	95% CI	*P*-value
Patients characteristics			
≤50 yrs	1	—	—
51–70 yrs	1.52	1.22–1.82	0.001
Age > 70 yrs	3.60	2.72–4.56	0.001
Female gender	1.70	1.42–2.16	0.001
Diabetes mellitus	1.78	1.51–2.15	0.001
Hypertension	0.92	0.80–1.15	0.38
Current smoking	0.83	0.63–0.96	0.08
Obesity	0.85	0.57–1.46	0.60
Medication during admission (1st 24 hrs therapy)			
*β*-blockers	0.28	0.23–0.34	0.001
Antiplatelet drugs	0.20	0.16–0.26	0.001
ACE/ARBs	0.37	0.30–0.48	0.001
Heparin	1.43	1.12–1.65	0.001
Thrombolytic	0.83	0.70–1.02	0.06
Revascularization procedures			
PCI	0.66	0.47–0.98	0.03
CABG	0.45	0.25–0.90	0.02

Adjusted OR: adjusted odd ratio; 95% CI: 95% confidence interval.
